# Self-Erasing Network for Person Re-Identification

**DOI:** 10.3390/s21134262

**Published:** 2021-06-22

**Authors:** Xinyue Fan, Yang Lin, Chaoxi Zhang, Jia Zhang

**Affiliations:** 1 School of Communication and Information Engineering, Chongqing University of Posts and Telecommunications, Chongqing 400065, China; fanxy@cqupt.edu.cn (X.F.); s190101035@stu.cqupt.edu.cn (C.Z.); s190131231@stu.cqupt.edu.cn (J.Z.); 2 Sichuan Provincial Key Laboratory of Intelligent Terminal Jointly Built by Departments and Cities, Yibin University of Electronic Science and Technology Research Institute, Yibin 644000, China

**Keywords:** person re-identification, deep learning, background suppression, maximum activation suppression

## Abstract

Person re-identification (ReID) plays an important role in intelligent surveillance and receives widespread attention from academics and the industry. Due to extreme changes in viewing angles, some discriminative local regions are suppressed. In addition, the data with similar backgrounds collected by a fixed viewing angle camera will also affect the model’s ability to distinguish a person. Therefore, we need to discover more fine-grained information to form the overall characteristics of each identity. The proposed self-erasing network structure composed of three branches benefits the extraction of global information, the suppression of background noise and the mining of local information. The two self-erasing strategies that we proposed encourage the network to focus on foreground information and strengthen the model’s ability to encode weak features so as to form more effective and richer visual cues of a person. Extensive experiments show that the proposed method is competitive with the advanced methods and achieves state-of-the-art performance on DukeMTMC-ReID and CUHK-03(D) datasets. Furthermore, it can be seen from the activation map that the proposed method is beneficial to spread the attention to the whole body. Both metrics and the activation map validate the effectiveness of our proposed method.

## 1. Introduction

Person re-identification is a task that uses computer vision technology to retrieve the identity of a specific person in surveillance with multiple non-overlapping cameras. This technology can be widely used in personalized patient monitoring, intelligent shopping malls and large-scale personnel search in video surveillance. Although the performance of ReID continues to improve with the help of deep convolutional neural networks, it still faces many challenges, e.g., various background clutter, light changes, occlusions and changes in attitude, which will affect the model feature coding.

**Figure 1:**
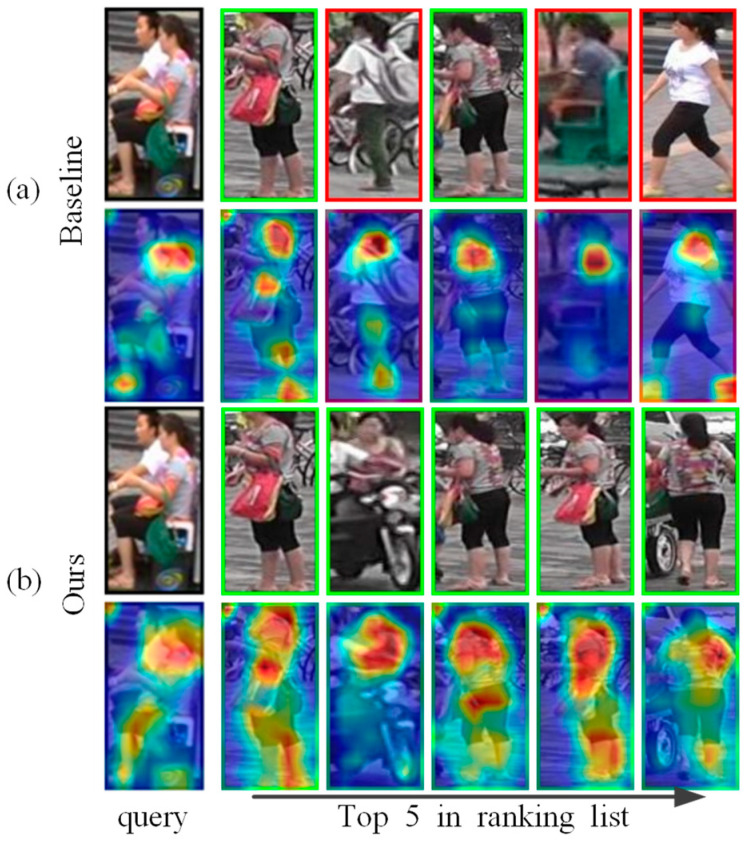
(**a**,**b**) represent the top 5 query results returned by the same query image through the baseline and the proposed method. The images with the green and red boxes are positive results and negative results. We also show the activation maps of each image; the second line shows that the baseline focuses on the person’s torso. The difference is that the proposed method discovers more visual cues from the whole body, as shown in the 4th line.

Due to person re-identification tasks that often encounter posture changes and background clutters, neural networks tend to focus on the main part of a person. Through visual analysis of the model, the baseline model tends to use a small number but distinguishable cues to identify a person. Although a few cues are sufficient to distinguish the identity of a person in the training set, when the environment becomes extremely complex, the robustness of the model is subjected to a tough test. In Figure 1, the baseline focuses on the head and feet of the query image and is disturbed by the man in white clothes in the background, so two women in white clothes appeared in the returned results. Our method further pays attention to the rich color features of the upper body of a person as well as the information of the legs, which makes the ranges of attention spread to the whole body, and the returned five query results are all correct. Therefore, the ReID model needs to find richer discriminative visual cues to form the comprehensive characteristics of each identity.

When viewing an object, the visual system always deliberately suppresses our uninterested region to find the most distinctive part; in this process, it can always unintentionally and successfully ignore the interference caused by the background, but the neural network itself does not have this function. With the person re-identification datasets collected by different cameras with a fixed angle of view, the background regions of the images collected under the same camera usually have some similarities. Moreover, the existence of similar clothes and postures will confuse the model, which prompts us to explicitly provide the network model with a roughly accurate background before restricting the observable region to the semantic fields.

To improve the performance of the model, a variety of research methods were proposed in recent years, which are roughly divided into the following categories: (1) Global feature representation learning—the literature [1,2,3] fully excavated the shallow high-resolution and deep high-semantic information and used global average pooling in different stages of the residual network to generate the embedding of each stage and obtained global information at different depths of the network. Qian et al. [4] designed a multi-scale deep learning model and used an adaptive method to find features of an appropriate scale for person retrieval; (2) Local feature representation learning—this commits to discovering more fine-grained visual information distributed throughout the human body. Zhang et al. [5] used the method of dynamic programming to calculate the local distance to realize the automatic alignment of the local features. The interaction-and-aggregation module [6] is designed to extract detailed features in the image and solve the problem of spatial position mismatch. Cai et al. [7] proposed a multi-scale body-part semantic mask guide attention network, which uses the semantic mask information of three parts of a person to guide the training of corresponding position attention. Second-order non-local attention [8] is introduced to strengthen the connection between multiple parts. Part-based Convolutional Baseline (PCB) [9] uses a refined part pooling strategy to make the feature closer to the real region; (3) Optimization strategy—some works attempted to achieve better person features with the modification of the backbone architecture, data enhancement, and regularization. The literature [2,3] changed the down-sampling convolutional stripe in the final stage of ResNet50 to 1. OSNet [10] introduced the residual block composed of multiple convolution streams to realize multi-scale feature learning. Zhong et al. [11] used a random erasure strategy to generalize the occlusion problem. AdaptiveReID [12] imposes a penalty on the objective function by adaptively updating the regularization factor to improve the robustness of the model. The Generative Adversarial Network (GAN) is adopted in [13] for data expansion.

**Figure 2:**
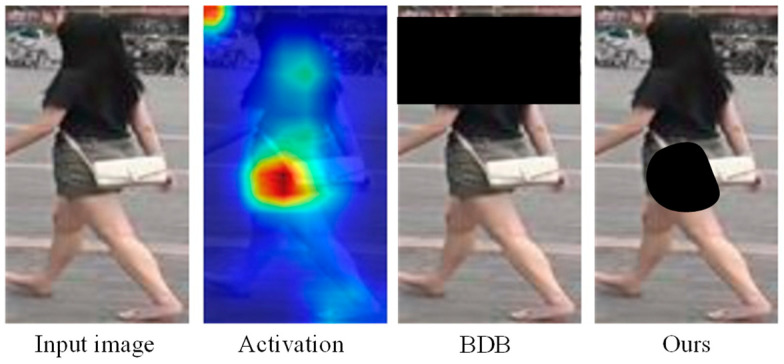
BDB creates a random block mask, while the proposed method creates an unshaped mask based on the most active part of the feature.

The proposed approach belongs to the second category in the above classification, which promotes the network to focus on the learning of task-relevant regions and encode weak features with distinguishing characteristics to discover the fine-grained features scattered over the whole human body. The self-erasing network contains three branches: (1) the global branch extracts the global features, and as a regularization method to solve the errors introduced by feature suppression; (2) the background suppression branch encourages the network to discover more high-quality features from the potential regions by erasing the low activated features, thus focusing on real semantic objects; (3) the maximum activation suppression branch forces the network to find more discriminative cues in the remaining weak information regions by erasing the most activated feature vector. We created an unshaped mask for each person based on the high activation regions of the feature as opposed to generating the random block mask as performed by the Batch Drop-Block (BDB) Network [14]. As shown in Figure 2, the activation regions are concentrated on the crotch of the person during training; if the BDB model randomly drops the upper body features, this will encourage the network to continue to focus on the crotch feature. While our method encourages the network to learn the upper body and leg information by occluding the crotch features, we believe that using the remaining features with lower recognition capabilities to stimulate the network can further discover more regions related to the target person. The results show that this learning strategy helps to extend the activation ranges to the whole body (Figure 1).

The contributions of this work can be summarized as follows:

(1) We designed an end-to-end multi-branch self-erasing network to overcome background interference and extract sufficient local information;
(2) Through the visualization of activation maps, we found that fully digging out a more comprehensive feature representation of a person helps to improve the discriminability of the model.
(3) Extensive experiments show that the proposed model has certain advantages over recent state-of-the-art methods.

## 2. Related Work

The concept of person re-identification was first proposed in 2006. In the early research work, hand-designed low-level visual feature extraction methods (e.g., color features, texture features and shape features) were difficult to cope with the challenges of complex scenes, which directly affect the accuracy of the metric learning. The prosperous deep neural networks introduce feature representations with better discrimination and robustness for person re-identification tasks, which push the performance of the model to a new level and make the end-to-end model that automatically extracts features become the mainstream.

To train an effective feature extractor to capture rich discriminative information, many methods integrate global features, local features and attention mechanisms. Harmonious Attention Network (HA-CNN) [15] jointly learns the soft pixel attention and hard regional attention and introduces a cross-sensing mutual learning mechanism to capture progressive cues from global branches to local branches. The Horizontal Pyramid Matching (HPM) approach is used in [16,17,18] to independently learn various partial information of a given person to enhance the discrimination of the overall characteristics, but the rigid space division undoubtedly increases the uncertainty. Some methods [19,20,21] use prior information related to a person’s nature (e.g., semantic attributes, pose, body parts) to reinforce the feature representation, but obtaining the corresponding semantic regions and body skeleton tags definitely increases the complexity of the task. Compared with the above methods, the proposed method can realize the autonomous suppression operation of features without additional positioning information. The attention modules are used in the most advanced person re-identification architecture [2,3,15,17], which assign different weights to the channels and spaces features, allowing the model to pay more attention to the important features of a person and suppress unnecessary interference. The above methods are dedicated to strengthening the encoding of the rich features in the input image, but due to the drastic visual angle changes, the features of the consistent salient regions may introduce errors, that is to say, the discriminative information that can be used to match two people may not be available in all situations. Therefore, the proposed method mines more abundant feature information from the less discriminative regions available by suppressing the maximum activation regions, which can still maintain the performance of the model without relying on the high saliency regions.

Some other methods use mask suppression to mine more comprehensive characterization information. Hou et al. [22] proposed SeeNet, which consists of two self-erasing strategies for semantic segmentation tasks, introducing the roughly accurate background priors to prohibit the attention regions from spreading to the background regions and allowing the network to find more semantic objects in the potential zone by erasing the attention regions. BDB [14] randomly erases the same region of the same batch of image features, allowing the model to find more valuable information in the remaining local region. A Mask-Guided Contrastive Attention Model (MGCAM) [23] was designed to generate a pair of body perception and background perception attention maps, which takes RGB data and a mask containing important body information as input at the same time, so as to learn features that are not disturbed by background clutter; however, MGCAM requires an additional Fully Convolutional Networks (FCN) [24] segmentation model in the preprocessing stage to generate a mask corresponding to the human body and the background. In contrast, our method is relatively simple, which forces the network to pay attention to the foreground information by directly discarding the low-confidence features generated in the final stage of the model.

## 3. Proposed Method

### 3.1. Network Architecture

**Figure 3:**
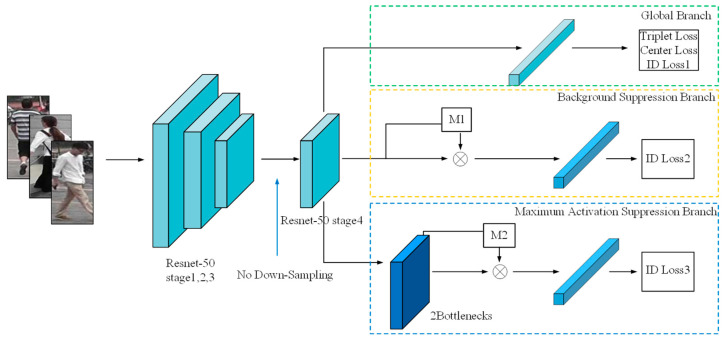
Proposed self-erasing network with three branches. The convolution step of the final stage of the backbone network is changed from 2 to 1 to retain more image information. The global branch sends the feature vector after global average pooling to the triplet loss, center loss and ID loss. The background suppression branch and the maximum activation suppression branch, respectively, use global average pooling and global maximum pooling for spatial dimensionality reduction and use ID loss for supervision, where all the features using ID loss are normalized.

We used ResNet50 as the backbone network for feature extraction, but with a slight modification, we did not use the down-sampling operation of stage4; in this way, we obtained a map size of 2048 × 16 × 8. In Figure 3, The first branch, known as the global branch, provides a global feature representation in the self-erasing network architecture. We obtained a 2048-dimensional feature vector by employing global average pooling and jointly used center loss and triplet loss to reduce the intra-class distance and increase the feature difference between classes; then, the 2048-dimensional feature vector is supervised by the ID loss after the BN layer. The second branch is the background suppression branch, which generates a background suppression mask M1 based on the output features of the backbone network, and performs dot multiplication with the output features; it reduces the interference caused by the background to a certain extent. We employed a global average pooling layer and a BN layer to obtain a 2048-dimensional feature vector. For the third branch, named the maximum activation suppression branch, the maximum activation suppression mask M2 is generated based on the output features of the two bottlenecks and multiplied with the output features, and then the global maximum pooling operation is used to solve the noise impact caused by M2 during feature dropping process. For the second and third feature suppression branches, a certain percentage of erasure ratios are used to generate suppression regions with different shapes for each ID, and it is ensured that the suppression regions of different channels of each image are the same. During the test, we used the concatenated features of the global branch and the maximum activation suppression branch to measure. Note that the feature of the maximum activation suppression branch does not pass through M2 at this time.

### 3.2. Self-Erasing Network

Existing models usually use global average pooling to reduce the dimensionality of the obtained features, but when down-sampling the overall feature information, it will largely retain the background information. Therefore, the proposed background suppression branch uses the generated M1 to suppress the background, enforcing the network to pay more attention to the foreground features. The maximum activation suppression branch adds two bottleneck layers behind the backbone network. By suppressing the most active continuous region through M2, the network is pushed to find more discriminative information in the remaining low information features. We tried to put M2 in front of the bottlenecks, due to the small resolution of the person dataset, the features after M2 introduces a lot of interference. We also tried to use the strategy in the semantic segmentation task, which constrains the third branch with a mask generated by the backbone network, this method is not applicable due to different tasks. Therefore, we followed the method of the BDB Network, which uses M2 after the bottleneck and directly sends the third branch features to the classifier for judgment.

#### 3.2.1. Background Suppression Branch

**Figure 4:**
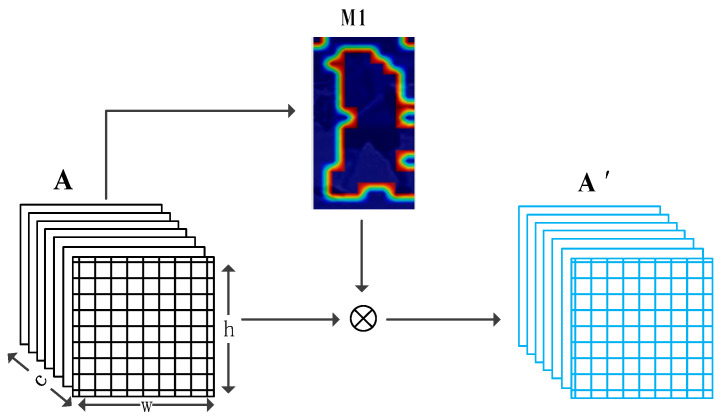
Illustration of M1 generation and deployment, where *A* represents the output feature of the backbone network, M1 is the background mask generated by *A*, *A’* is the feature after M1.

The background suppression branch does not need to be based on any annotated information and directly creates M1 based on the output of the features by the backbone network. As shown in Figure 4, assuming that b is the size of the training batch, c, h, and w represent the channel number, height and width of the feature, respectively. Each feature channel is equivalent to a discriminator to obtain different types of features, but their response ranges are roughly the same, so we suppressed the same ranges of different channels of the same person, which does not affect the feature representation of each channel. According to the definition of activation map by Zagoruyko et al. [25], first, the *c*-dimensional features are merged into one channel feature to form the activation map *F*.

(1) $$F_{}^{p}(A)={\sum_{i=1}^{C}\left|A_{i}\right|}^{p}$$

where *Ai* represents the feature tensor of size *h* × *w*. The larger the *p*, the more attention will be paid to the most distinctive parts, here *p* is set to 2. Then, we compressed the width dimension information and height dimension information into one dimension and generated feature *Z*.

(2) $$Z=Sorted(F_{}^{p}(A))$$

Finally, according to the deletion ratio *r_1_*, the smallest *N_1_* features are set to zero to generate a binary mask M1. The deletion ratio represents the ratio of the number of elements with zero to the total feature of size *h × w*.

(3) $$M1_{i=1\cdots c}=\left\{ \begin{array}{l} 0,if\;Z\in minest\;N_{1}\\ 1,otherwise \end{array}\right.N_{1}=r_{1}\times h\times w$$

For the background suppression process, we created a mask M1 of the same size as the output feature *A* for each ID, then expanded the generated mask into *c* channels and performed a dot product with the feature *A* to obtain the feature $A^{\prime }$.

(4) $$A^{\prime }=A⊙M1_{i=1\cdots c}$$

**Figure 5:**
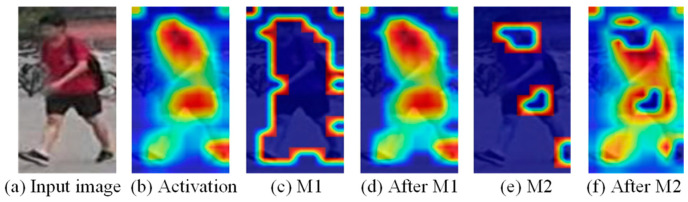
Activation maps, the background mask M1 and the maximum activation mask M2 of the input image, and the effects after M1, M2 treatment.

In Figure 5b, the activation ranges of the original image contain a large amount of background information, such as the lawn and bicycles behind the person. As mentioned already, we multiplied the generated M1 with the output feature to obtain the effect of Figure 5d. It can be seen that the contour of the activation map is clearer after removing the weak features. The model is forced to focus on the foreground of the human body and fully excavate the characterization information related to the character. We introduced the same erasure ratio for each ID feature map, which is equivalent to suppressing the fixed-size background data of each image. In the feature measurement, each feature map loses a certain percentage of features, so the judgment for each person is fair. Finally, we employed global average pooling on the masked features, the global information obtained in this way is more effective after overcoming background interference. Luo et al. [26] found that the gradient descent direction of the triplet loss and the ID loss is different, and the constraining ability of the ID loss is better than the triplet loss, so we only used the ID loss for the supervision of the background suppression branch. Due to the existence of the global branch, the noise caused by the wrong background erasure region at the initial training stage can be effectively monitored.

#### 3.2.2. Maximum Activation Suppression Branch 

After background suppression, the model focuses on the foreground of the character, which overcomes the interference brought by the background. The experiment found that the attention of the network at this time is too focused on the person’s torso (Figure 1a), ignoring the distinguishing local region. Inspired by the activation in the second row of Figure 1a, we proposed to extend the activation ranges of the baseline model. The maximum activation suppression branch overcomes the problem of the model’s attentional regions overly focusing by removing the most active indefinite shape region in the feature and maintaining the discriminability of the network by learning multiple scattered feature regions with rich semantics. Similar to the background suppression branch, we suppressed the most activated parts with a fixed erasure ratio. During training, the global branch and background suppression branch directly extract global features from the backbone network and can handle the noise interference caused by the discarded features of the maximum activation suppression branch. The maximum activation suppression branch also serves as a global auxiliary branch; the two bottlenecks behind the backbone network can more fully extract local information. Note that the global maximum pooling is used in this branch because the global maximum pooling encourages the network to find more discriminative information in the remaining weak features, thereby enhancing the model’s ability to distinguish persons by using low- discriminative features.

The generation and deployment of the mask are the same as the background suppression branch, but the difference is that the most activated parts of the feature after the two bottlenecks are suppressed here. In Figure 4e, the generated mask M2 can accurately suppress the person’s head, waist, and feet so that the network can find a more comprehensive person representation in the features after M2.

(5) $$M2_{i=1\cdots c}=\left\{ \begin{array}{l} 0,if\;Z\in largest\;N_{2}\\ 1,otherwise \end{array}\right.N_{2}=r_{2}\times h\times w$$

### 3.3. Loss Function

We employed three types of loss functions in the self-erasing network, each branch has a cross-entropy loss [27] as ID loss, and the global branch adds a center loss [28] and a triplet loss [29].

Smoothing Cross-Entropy Loss: Since the same image of the person does not appear in the training set and the test set at the same time, and through visualization, it was found that there were a small number of errors in the labels. Therefore it is particularly important for the ReID task to prevent the model from overfitting the training set. Label smoothing is an effective way to alleviate overfitting in classification tasks, and its formula can be defined as:

(6) $$q_{i}=\{\begin{array}{ll} 1-\frac{N-1}{N}\varepsilon & if\;i=y\\ \frac{\varepsilon }{N} & otherwise \end{array}$$

where i∈1,2,⋯,N represents the sample category, y represents truth ID label, *ε* is a constant indicating the degree to which the model does not trust the training set, we set it to 0.1 in the experiment. After smoothing by the above formula, the existing non-zero or one labels are slightly offset. The cross-entropy loss function is formulated as follows:

(7) $$L_{id}=\sum_{i=1}^{N}-q_{i}log(p_{i})$$

where $p_{i}$ represents the predicted probability of class *i*, $q_{i}$ represents the truth label.

Triplet loss: We used the sampled hard triplet loss to train the global branch. The loss function can be written as:

(8) $$L_{tri}=\frac{1}{P\times K}\sum_{a\in batch}max(\underset{p\in A}{max}d_{a,p}-\underset{n\in B}{min}d_{a,n}+\alpha ],0)$$

In each training batch, there are *P* persons, and each person has *K* different images. $d_{ap}$ and $d_{an}$ represent the distance of the positive samples pair and negative samples pair, respectively. The so-called hard sample is to use each image as an anchor a to find the hardest positive sample *p* (the farthest distance to *a*) and negative sample *n* (the closest distance to *a*) in a batch. α is a constant used to stretch the distance between $d_{ap}$ and $d_{an}$.

Center loss: To compensate for the triplet loss only considers the relative distance between $d_{ap}$ and $d_{an}$, and ignores the absolute distance, we used the center loss to minimize the intra-class distance to increase the intra-class compactness, improving the distinguishability between features, formulated as:

(9) $$L_{c}=\frac{1}{2}\sum_{i=1}^{batch}{\left\|f_{i}-c_{i}\right\|}^{2}$$

where fi is the feature of the *i*-th class, $c_{yi}$ is the feature center of the *i*-th class. The total loss of our model is as follows:

(10) $$L_{}=L_{ID1}+\lambda_{1}L_{ID2}+L_{ID3}+L_{tri}+\lambda_{2}L_{C}$$

where $\lambda_{1}$ and $\lambda_{2}$ are the weight coefficient, set to 0.5 and 0.0005, respectively.

## 4. Experimental Results

### 4.1. Implementation Details

All our experiments were employed on a single RTX 2080 Ti (10 GB GPU memory). Resnet-50 was used as the backbone network and initialized with ImageNet pre-trained parameters. During training, the input images were resized to 256 × 128 and augmented by random horizontal flip with *p* = 0.5 and random erasing with *p* = 0.5. A training batch of 64 images contained 16 IDs, and each ID had 4 images. The background erasure ratio and the maximum activation erasure ratio were set to 0.1 and 0.25. We used the Adam optimizer with the initial learning rate of $4.5\times 10^{-5}$ to optimize the five losses, and the following learning rate decay was as follows.

(11) $$lr(t)=\left\{ \begin{array}{ll} 4.5\times 10^{-5}\times \frac{t}{10} & if\;t\leq 10\\ 4.5\times 10^{-4} & if\;10<t\leq 45\\ 4.5\times 10^{-5} & if\;45<t\leq 90\\ 4.5\times 10^{-6} & if\;90<t\leq 210 \end{array}\right.$$

### 4.2. Datasets and Evaluation Metrics

#### 4.2.1. Datasets

In Table 1, we analyzed the commonly used Market1501 [30], DukeMTMC-ReID [31] and CUHK03 [32] datasets from the six perspectives: training ID, training image, gallery ID, gallery image, camera number and image resolution, and verified the proposed method in these datasets.

Market-1501 contained 1501 identities collected by 6 cameras at Tsinghua University, of which the training set had 12,936 images of 751 identities, and another 750 identities were used for testing, including 3368 query images and 19,732 gallery images.

DukeMTMC-ReID was collected by 8 cameras with various resolutions and contained 16,522 training images with 702 identities. The query set contained 2228 images with 702 identities, and the gallery set, which added 408 interference items, contained 17,761 images with 1110 identities.

CUHK03 contained 1467 identities collected by 10 cameras, of which 767 identities were used for training, and another 700 identities were used for testing. It was divided into CUHK03(L) and CUHK03(D) according to the label generation method. CUHK03(L) contained 7368 training images, 1400 query images, 5328 gallery images, and CUHK03(D) contained 7365 training images, 1400 query images, 5332 gallery images.

#### 4.2.2. Evaluation Metrics

We used cumulative match characteristic (CMC) and the mean average precision (mAP) metrics to evaluate the quality of person re-identification models.

CMC: The horizontal coordinate of the CMC curve represents rank1 to rank-k, and the vertical coordinate represents the matching rate. Among them, rank-k represents the ratio of the number of test samples correctly identified by the model to the total number of test samples in the first k result sequences. Rank-1 represents the first hit rate of the model, which is of more reference significance.

mAP: Unlike CMC, which only cares about the ranking of the top positive samples in the results sequences, *mAP* is determined by the ranking results of all positive samples, so it can better reflect the robustness of the model. *AP* represents the average precision of the model’s retrieval of a single image; its formula can be written as:

(12) $$AP=\frac{1}{N}\sum_{i=1}^{N}i/Mi$$

where *N* represents the number of images that are correctly matched to the query image in the gallery, *Mi* represents the number of images retrieved in total when the *i*-th image is correctly retrieved. *mAP* is the average of *AP*, which is used to evaluate the search ability of the model for all query images; its formula is as follows:

(13) $$mAP=\frac{1}{C}\sum_{k=1}^{C}AP(k)$$

where *C* represents the number of the query set. Since the above two metrics may show different preferences, we averaged them and rounded them to one decimal place to obtain the score, which is added as an evaluation indicator.

### 4.3. Ablation Study

We evaluated the effectiveness of the self-erasing network on three datasets. Baseline represents the global branch deployed on the backbone network. BS and MS represent the background suppression branch and maximum activation suppression branch, respectively. As shown in Table 2 that the performance of the BS branch improved slightly on the Market1501 and CUHK03(L). On DukeMTMC-ReID, Rank-1 and mAP increased by 1.7% and 1.2%. On CUHK03(D), Rank-1 and mAP increased by 2.1% and 1.2%, respectively. There was a significant improvement of the BS branch on the DukeMTMC-ReID dataset with more severe background occlusion, which indicates that the BS branch promotes the backbone network to encode information related to the person’s foreground from the input image.

The maximum activation suppression branch encourages the backbone network to discover more relevant local features and enhances its ability to mine more discriminative information under the supervision of weak features. When the baseline adds the MS branch, the two indicators increased by about 1% on the Market1501 and CUHK03(L). The MS branch can increase the Rank-1 and mAP by 1.9% and 1.5% on DukeMTMC-ReID, and increase the Rank-1 and mAP by 3.4% and 3.3% on CUHK03(D).

**Figure 6:**
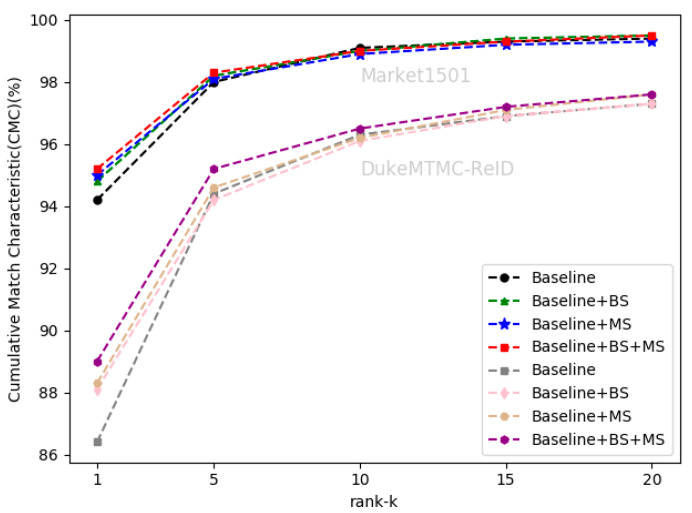
The CMC curve on Market1501 and DukeMTMC-ReID.

**Figure 7:**
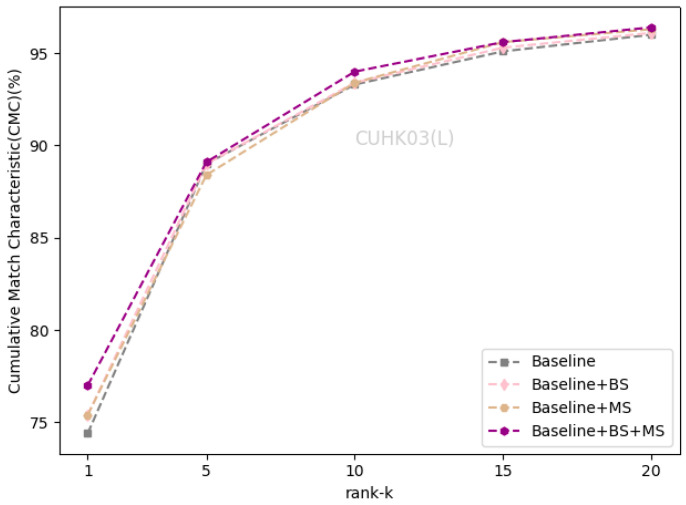
The CMC curve on CUHK03(L).

**Figure 8:**
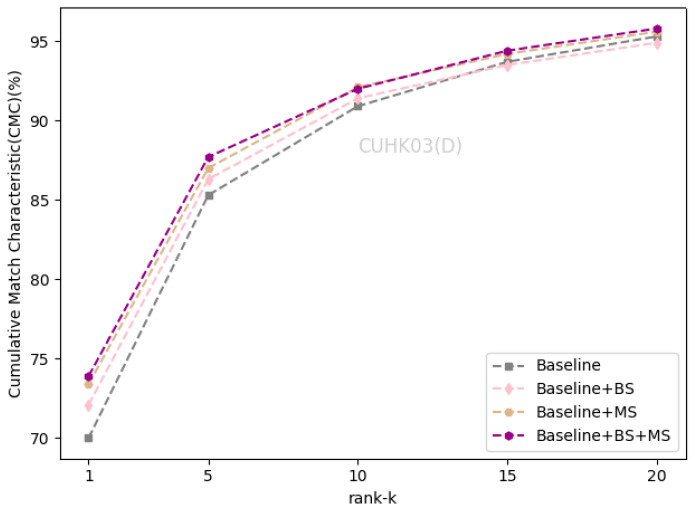
The CMC curve on CUHK03(D).

**Figure 9:**
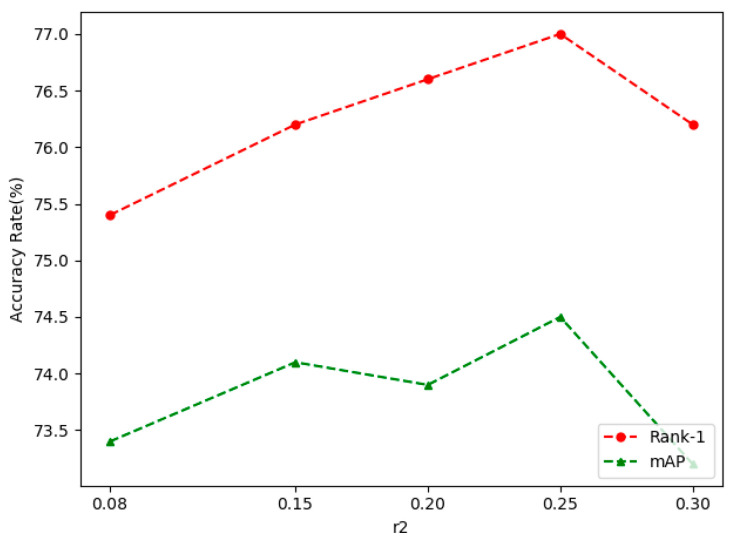
The effect of r2 on model performance.

After completing the fusion of the two branches, the performance of the self-erasing network on three datasets was significantly improved. The most prominent was the increase of mAP and Rank-1 by 3.9% and 3.2% on CUHK03(D). Figure 6, Figure 7 and Figure 8 show the CMC curve of the proposed method on the three datasets: Market1501, DukeMTMC-ReID and CUHK03. From these graphs, we can see that our method has brought a huge improvement in the model performance. In Figure 9, we show the effect of the erasure ratio on the performance of the self-erasing network on CUHK03(L). Here, we set the background erasure ratio(r1) to 0.1 due to a large number of weak features in the images and then changed the most activated erasure ratio(r2). It can be seen that when r2 is 0.25, both Rank-1 and mAP achieve the best results.

In addition, we analyzed the process of gradually discovering more fine-grained information under the self-erasing network at different stages of training and compared the evolution of activation maps for baseline and self-erasing networks at different epochs. Here we will show more clearly how our method encourages the network to pay attention to foreground information and fully excavate more detailed local regions to complete the improvement of model performance.

**Figure 10:**
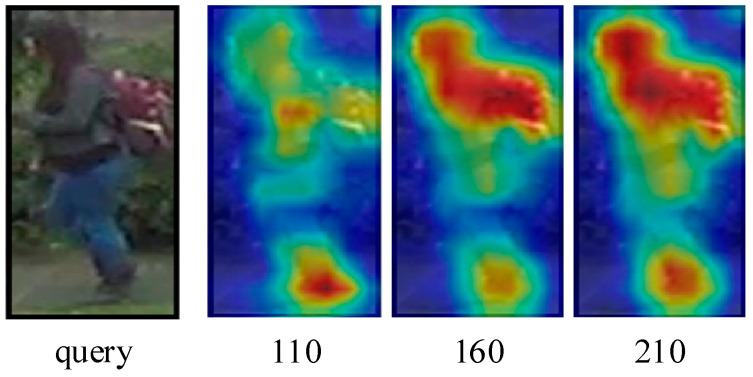
Evolution of the activation maps at different epochs.

There is a great deal of occlusion and serious background interference in the DukeMTMC-ReID dataset. What we show in Figure 10 is a very low-resolution query image under its dataset, and the woman’s purple jacket is confused with the background. Through the self-erasing network, the model can still roughly focus on the person’s whole body at 110th epochs, but as with the baseline, the model tends to focus more on the person’s torso, such as the feet. Then at 160 epochs, the most activated foot is suppressed, and the model’s attention is forced to turn to other parts with discriminatory cues, e.g., colored backpacks and the upper body. At 210th epochs, the activation regions of the network are scattered all over the person and focus on the person’s head, upper body, backpack, and feet, forming the overall characteristics of each identity. This is in line with our original design goal, which uses rich discriminative visual cues to make the model better.

**Figure 11:**
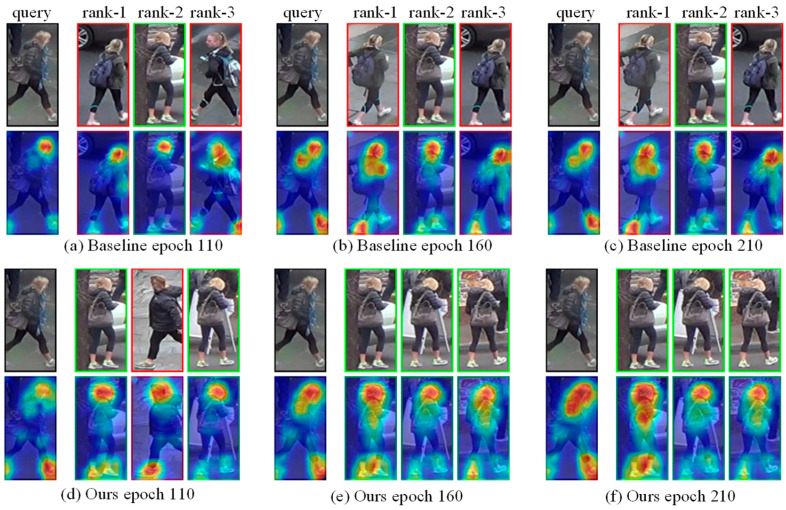
The ranking results and activation maps of baseline and self-erasing network at different epochs. Images with the green and red boxes are positive results and negative results.

In Figure 11, we compare the search capabilities (rank1–rank3) of the self-erasing network and the baseline model for query images at different epochs and show the evolution of the activation maps. Similarly, we selected a difficult query image in the DukeMTMC-ReID. It can be seen from the image that the color of the query image is dark, the light is insufficient, and the appearance of the person is similar to the background. Therefore, at 110th epochs, the activation regions of the baseline and our method mainly exist in the head and foot parts of the query person. In contrast, our method has deepened the learning of back and leg information, so two of the returned results are correct. At 160th epochs, the baseline model has a certain improvement in the activation regions compared to the previous epochs. Judging from the returned error results, similar features (e.g., white shoes, gray tops, blond hair) are a huge challenge to the model. The activation regions of our network basically spread to the whole body and strengthen the learning of key parts, and the returned results are all correct. At 210th epochs, the attention regions of the baseline and our method change slightly. Through the above analysis, our model can still maintain good performance under very similar characteristics.

### 4.4. Comparison with State-of-the-Art

We compared our work with the most advanced methods released in 2019 and 2020 on the Market1501, DukeMTMC-ReID, and CUHK03 datasets. All reported results have not undergone re-ranking operations.

Table 3 lists results on Market1501 and DukeMTMC-ReID with the best metrics bonded. On Market1501, our method underperforms DSA-reID [33] by 0.5% in Rank-1, but still gets the same score with DSA-reID [33] and CLFA [34]. Compared with MVP [35], we improve Rank-1 and mAP by 3.8% and 7.6%, respectively. On DukeMTMC-ReID, our method achieves 89% and 78.5% accuracy on Rank-1 and mAP, respectively, which exceeds all compared methods.

Table 4 lists results on CUHK-03 with the best metrics bonded. On CUHK-03(L), the total score of our method is 0.2% less than the first-best P2-Net [36], but it still outperforms CAMA [37] by 7.5%. Our method also achieves the best ranking on CUHK-03(D) with 73.9% Rank-1 and 71% mAP, respectively. In general, the self-erasing network is very competitive in the methods compared above.

### 4.5. Limitations

The proposed model presents two limitations: small targets and occlusions. These two problems affect the model’s feature extraction ability and reduce the accuracy of retrieval. The small targets refer to the small proportion of person in the image, so there will be a lot of interference information, which increases the difficulty and uncertainty of global branch feature extraction and indirectly affects the mining of local branch multi-granularity information. Regarding the occlusions, because the designed model does not specifically use key point positioning to obtain more prior knowledge, when people are severely occluded, the information that can be used for mining is very limited, which is a challenge for the maximum activation suppression branch. The above two problems are also to be solved in future work. Overall, considering the good performance indicators and simple model design of our proposed model, we believe that our work will have a certain value for the ReID field and other computer vision fields.

### 4.6. Future Work

In future work, we plan to deploy our strategy on other small networks to make it more in line with the purpose of small engineering costs. Additionally, we intend to adopt the method of key point positioning to dig more features in a smaller local region and use the formed more comprehensive representation to improve the accuracy of the model’s retrieval.

## 5. Conclusions

In this paper, we introduce a simple yet powerful self-erasing network composed of three branches. The global branch aims to extract the global characteristics of a person. The background suppression branch overcomes background interference and allows the model to focus on the learning of the person foreground. The activation suppression branch strengthens the model’s ability to mine low-information regions and expands the activation ranges to obtain more extensive visual cues. The experimental results on three large datasets show the reliability of the proposed method, and we achieve the state-of-the-art performance on DukeMTMC-ReID and CUHK-03(D) datasets. Besides, the activation map further shows that our method improves the accuracy of model retrieval by mining more comprehensive distinguishing features instead of a single global feature, which provides some insights for the design of the person re-identification model.

## Figures and Tables

**Table 1:** Statistics of datasets.

Dataset	Training ID	Training Image	Gallery ID	Gallery Image	Camera	Resolution
Market1501	751	12936	750	19732	6	fixed
DukeMTMC-ReID	702	16522	1110	17661	8	vary
CUHK03(D)	767	7365	700	5332	10	vary

**Table 2:** Influences of self-erasing network on three datasets.

Method	Market1501		DukeMTMC-ReID		CUHK03(L)		CUHK03(D)	
	Rank-1	mAP	Rank-1	mAP	Rank-1	mAP	Rank-1	mAP
Baseline	94.2	86.2	86.4	76.4	74.4	72.7	70	67.9
+BS	94.8	87.2	88.1	77.6	75.4	73.1	72.1	69.1
+MS	95	87.5	88.3	77.9	75.4	73.4	73.4	71.2
+BS+MS	95.2	88.1	89	78.5	77	74.5	73.9	71

**Table 3:** Comparison of different methods on Market1501 and DukeMTMC-ReID.

Method	Source	Market1501			DukeMTMC-ReID		
		Rank-1	mAP	Score	Rank-1	mAP	Score
MVP [35]	ICCV2019	91.4	80.5	86	83.4	70.0	76.7
CAMA [37]	CVPR2019	94.7	84.5	89.6	85.8	72.9	79.4
Auto-ReID [38]	ICCV2019	94.5	85.1	89.8	88.5	75.1	81.8
OSNet [10]	CVPR2019	94.8	84.9	89.9	86.6	74.8	80.7
DGNet [39]	CVPR2019	94.8	86	90.4	86.6	74.8	80.7
DSA-reID [33]	CVPR2019	**95.7**	87.6	**91.7**	86.2	74.3	80.3
HOReID [40]	CVPR2020	94.2	84.9	89.6	86.9	75.6	81.3
CLFA [34]	CVPR2020	95.4	88.0	**91.7**	88.3	**79.1**	83.7
Ours	-	95.2	**88.1**	**91.7**	**89**	78.5	**83.8**

**Table 4:** Comparison of different methods on CUHK-03.

Method	Source	L			D		
		Rank-1	mAP	Score	Rank-1	mAP	Score
Auto-ReID [38]	ICCV2019	73.0	**77.9**	75.5	73.3	69.3	71.3
CAMA [37]	CVPR2019	70.1	66.5	68.3	66.6	64.2	65.4
P2-Net [36]	ICCV2019	**78.3**	73.6	**76**	**74.9**	68.9	71.9
OSNet [10]	CVPR2019	-	-	-	72.3	67.8	70.1
MHN [41]	CVPR2019	77.2	72.4	74.8	71.7	65.4	68.6
CLFA [34]	CVPR2020	76.3	74.5	75.4	72.3	70.3	71.3
Ours	-	77	74.5	75.8	73.9	**71**	**72.5**

## Abbreviations

**Acronyms**	**Full Form**
BDB	Batch Drop-Block
FCN	Fully Convolutional Networks
GAN	Generative Adversarial Network
HA-CNN	Harmonious Attention Network
HPM	Horizontal Pyramid Matching
MGCAM	Mask-Guided Contrastive Attention Model
PCB	Part-based Convolutional Baseline
ReID	Person re-identification
